# Possibility of Using Natural Zeolite Waste Granules Obtained by Pressure Agglomeration as a Sorbent for Petroleum Substances from Paved Surfaces

**DOI:** 10.3390/ma15196871

**Published:** 2022-10-03

**Authors:** Ewelina Pabiś-Mazgaj, Paweł Pichniarczyk, Agata Stempkowska, Tomasz Gawenda

**Affiliations:** 1 Glass and Building Materials Center, Łukasiewicz Research Network Institute of Ceramics and Building Materials, Cementowa 8 Str., 31-983 Cracow, Poland; 2 Faculty of Mining and Geoengineering, Department of Environmental Engineering, AGH University of Science and Technology, Mickiewicza 30 Av., 30-059 Cracow, Poland

**Keywords:** sorption, waste zeolite, agglomeration, roller press

## Abstract

Increasing incidents of oil spills and dynamic development of civilization are driving the demand for sorbents. The production of the overwhelming majority of mineral sorbents involves the highly energy-consuming calcination process with CO_2_ emissions impacting the environment. Taking into account the environmental issues related to greenhouse gas emissions, we are in urgent need of green products and green technologies. The aim of this study was to investigate the possibility of using natural zeolite dust waste to produce oil sorbents in non-calcination technology. The main advantage of the proposed solution is reduction of greenhouse gas emissions and transformation of the waste material into green, useful product. The scope of the research covered the experimental tests for compaction of the zeolite material from Ukraine and Slovakia in a roller press with different type of binding agent and the performance tests for assessing the suitability of the produced agglomerates as a petroleum sorbent. In order to evaluate the agglomerates’ properties, textural-structural analysis (optical microscopy, SEM microscopy, and low-temperature N_2_ sorption), petroleum sorption (Westinghouse method), and physical-mechanical tests (resistance to gravitational drop, abrasion resistance) were carried out. Properties of the manufactured agglomerates were studied in comparison to commercial sorbent DAMSORB, commonly used in Poland. The test results showed that it is doable to produce the effective surface oil-spill sorbent from zeolite waste dust in a roller press by applying the relevant binding agent. The main finding of this study was that optimum feed composition for dry granulation that provides the granular sorbent with the best properties was established: 6% of C binder and moisture content within a narrow range of 20–20.6%. The C-1 sorbent achieved the highest and closely comparable to commercial sorbent parameters of petroleum sorption and drop strength, which are key points of this study. The absorption efficiency of petroleum substances for C-1 sorbent is 8% lower than that of a commercial sorbent, and resistance to gravitational drop is lower by only 3%. However, commercial sorbent exhibited better abrasion resistance compared to produced agglomerates, which is a compelling reason to continue the research in order to enhance the abrasion performance of the manufactured granules. The effectiveness of the dust consolidation method carried out was proved by textural parameters of the obtained zeolite-based sorbents. Specific surface area (S_BET_) of B-2 (S_BET_ = 28.1 m^2^/g) and C-2 (S_BET_ = 28.3 m^2^/g) sorbents was very similar to the commercial sorbent (S_BET_ = 28.1 m^2^/g). Interestingly, all granules produced from clinoptilolite dust from Slovakia (A-2, B-2, C-2) achieved an increase of 19%, 33%, and 35%, respectively, in volume of mesopores, compared to the commercial sorbent. Moreover, the presented granulation technology favorably affected the size of the specific surface area as well as volume and surface area of mesopores in the case of obtained zeolite-based sorbent A-1 and B-1, compared with the starting raw material. Zeolite-based sorbent A-1 and B-1 achieved an increase of 17% and 18%, respectively, in specific area surface and an increase of 35% and 12%, respectively, in mesopores volume, compared with the raw material. Finally, the results of this investigation proved that it is possible to produce the efficient oil sorbent in a much more eco-friendly and green way, compared with that of the commercial sorbent.

## 1. Introduction

The dynamic development of technology, motorization, and the continuous progress of civilization indicate that the demand for sorbents of petroleum substances will continue to increase. Particularly dangerous and difficult to neutralize are uncontrolled leaks of petroleum compounds due to accidents, which have led to many environmental disasters [1,2]. Examples include, considered the largest environmental disaster in Sri Lanka’s history, the fire and sinking of an oil tanker in 2021, or the oil spill off the coast of Mauritius in 2020 [3]. Many times, the effects of environmental disasters caused by oil leaks are irreparable, and their consequences can have a lasting negative impact on the ecosystem. It should be remembered that spills of oil compounds, especially on land, can easily spread to further territories causing contamination of soil, groundwater, and surface water, among others. Therefore, the rapid and effective neutralization of oil spills through the use of appropriate deactivating agents is crucial in preventing serious consequences of ecosystem contamination [4]. There are four main methods of oil spill cleanup: adsorption methods, mechanical methods, and chemical and biological methods. The selection of a method for eliminating a hazardous substance spill depends on the cause of the spill and the extent of the spill. Usually, several methods are used simultaneously, with adsorptive methods using various adsorbents being the most common [5]. The use of sorbents in the disposal of spills of petroleum substances is considered the most effective, accessible, and inexpensive method. The main disadvantages of using sorbents are mentioned as the cost of disposing of used materials and the limitations in using sorbents for oil spills on water due to the tendency of most sorbents to sink in the aquatic environment [6]. The materials used in the removal of pollutants are divided into inorganic mineral sorbents, natural organic sorbents, and synthetic organic sorbents [5,6]. Of the group of synthetic sorbents, polypropylene [7] and polyacrylic [8] sorbents and organically modified synthetic zeolites [9] are the most commonly used for cleaning up oil spills. On the other hand, from the group of inorganic mineral sorbents, vermiculite [10], montmorillonite (commercial name: bentonite) [5], and diatomite [11] are used. In Poland, inorganic mineral adsorbents, among others, calcined diatomite obtained by high-temperature thermal treatment, are most often used to deactivate leaks of petroleum substances from paved surfaces. Natural zeolites are categorized as inorganic mineral adsorbents just like diatomite, diatomaceous earth, perlite, clay minerals, fly ash, and activated carbons [12]. Currently, a lot of research work is focused on the development of natural organic sorbents from various types of agricultural products such as corn stalk, cotton, nettle fiber orange peel, palm fiber, pineapple leaves, pomelo peel, rice husks, sawdust, wheat straw, sugarcane pomace, and walnut shells [9]. Zeolites are microporous aluminosilicates of the spatial type whose skeleton is made up of interconnected [SiO4]^−4^ and [AlO4]^−5^ tetrahedra. The ratio of silicon to aluminum in the spatial bonds of the tetrahedra varies depending on the type of zeolite mineral, with the silicon content always being higher [13]. In the crystal structure of zeolites, there is a system of channels and chambers of well-defined molecular size, which under standard conditions are filled with cations and water molecules with high mobility, the so-called zeolitic water [14]. The arrangement and size of the channel system in the zeolite framework is important because of the sorption and catalytic properties of zeolites [13]. Due to their specific physicochemical properties, zeolites have comprehensive possibilities of various applications. This type of mineral plays a crucial role in, among others, environmental protection (water, sewage, and gases purification or pollutant removal), agriculture, manufacturing, and industrial processes as well as in medicine as biomarkers detectors of various diseases or as antimicrobial materials [15,16,17]. Not without reason, zeolites are determined as the raw materials of the 21st century [15,18]. Considering growing demand for green building materials, zeolites can contribute to the production of the sustainable composite materials. Many studies [19,20,21,22,23,24,25] have proven that addition of natural zeolites in appropriate dosage to concrete composites can significantly improve the performance of the final concrete product and provide the environmentally friendly products. Ahmadi and Shekarchi [24] indicated that the application of 20% zeolite powder as a cement replacement can improve the comprehensive strength by 25%. Enhancement in compressive strength, permeability, and chemical durability of the concrete with zeolites as additives is mostly related with excellent pozzolanic properties of natural zeolites, especially zeolitic tuffs [26,27,28]. Markiv et al. [21] proved that zeolite can enhance the resistance to freezing and thawing damage and decrease drying shrinkage or water penetration in concrete, whereas other researchers [25] demonstrated that natural zeolites reduce chloride ion penetration into concrete. More recently, Ahdal et al. [29] investigated the mechanical and durable properties of concrete with addition of natural zeolites and waste PET plastic fibers as the cement replacement. As a result, researchers [29] produced the green concrete with addition of the 15% zeolites and 1% PET as the cement replacement. Test results [29] showed that zeolite- and PET-based concrete has better compressive strength compared with the control sample. In another study [30], the powder zeolite was used in production of innovative lightweight self-compacting cement composites. Adhikary et al. [30] applied the zeolite as mineral admixtures in order to improve the workability and thermal conductivity of the composite. Moreover, test results [30] proved that zeolite-added composites exhibited better strength parameters than fly-ash added composite. Zeyad et al. [31] proved that natural zeolite powder as waste product from the volcanic aggregate industry is a promising source of aluminum and silica for producing the hybrid geopolymer concrete. Researchers [31] demonstrated that volcanic pumice dust waste used as ordinary Portland cement or cement kiln dust replacement in amount of 30% can enhance the compressive strength and decrease the water absorption of geopolymer concrete

Based on their origin, we can divide zeolites into natural and synthetic. Today, the most popular are synthetic zeolites which have been successfully produced under laboratory conditions for many years [32]. It is also emphasized that synthetic zeolites have better physical and chemical properties than natural zeolites. However, it should be remembered that the manufacture of synthetic zeolites generates additional costs and involves the emission of harmful substances into the environment. In addition, in most cases, the developed methods of zeolite synthesis are applicable only in laboratory conditions, and their adaptation on an industrial scale is not always cost-effective. Natural zeolites show selectivity with respect to the size of absorbed particles and with respect to their polarity [33]. Zeolites have a negative electric charge on their surface, therefore they readily adsorb cations, while the ability to adsorb metallic anions or organic compounds is relatively weak. In terms of sorption of petroleum substances, which are non-polar substances, natural zeolites exhibit relatively poor sorption, although by chemical activation of zeolites, their sorption properties to petroleum substances can be effectively increased. Research on the use of zeolite as a sorbent of oil substances has been the subject of several works mainly in terms of the use of synthetic zeolites as sorbents of oil substances from water surfaces [1,34,35,36,37]. Regarding the use of zeolites for sorption of petroleum substances from paved surfaces, the literature is not extensive and there is a need for further research in this area [13]. Therefore, considering the literature gap, the purpose of this study was to determine the possibility of using zeolite granules obtained by pressure agglomeration of the zeolite dust waste as a sorbent for petroleum substances from paved surfaces. The research was divided into two stages. The first stage consisted of experimental studies to produce zeolite agglomerates differing in the origin of the zeolite waste material (clinoptilolite dust from Ukraine and clinoptilolite dust from Slovakia) and the type of binding agent used. The second stage consisted of evaluating the properties of the produced agglomerates for use as a sorbent for petroleum substances. For this purpose, textural-structural tests (optical microscopy, SEM microscopy, and low-temperature N_2_ sorption), tests of sorption of petroleum substances (Westinghouse method), and physical-mechanical tests (resistance to gravitational discharge, abrasion resistance) were carried out. The properties of the produced agglomerates were compared with those of the commercial sorbent of petroleum substances DAMSORB commonly used in Poland. DAMSORB is diatomite-based sorbent obtained in highly energy-consuming technology.

This research also aims to develop more environmentally friendly technology for sorbent production by using the waste materials and cutting greenhouse gases emissions during manufacturing. All of the produced zeolite-based sorbents achieved the minimum oil absorbency for sorbents used by fire departments in Poland, that is 50% by weight of the sorbent. Ukrainian zeolite-based sorbent with binder C exhibited the highest oil and water sorption in range of 76% and 88%, respectively. The achieved petroleum substance absorption rate is just 8% lower than for commercial sorbent DAMSORB. In addition, the mechanical strength in terms of gravitational drop rate decreases just by 3%, compared with that of the commercial sorbent. The effectiveness of the dust consolidation method carried out was proven by textural parameters of the obtained zeolite-based sorbents. Specific surface area (S_BET_) of B-2 (S_BET_ = 28.1 m^2^/g) and C-2 (S_BET_ = 28.3 m^2^/g) sorbents were very similar to the commercial sorbent (S_BET_ = 28.1 m^2^/g). Moreover, applied press granulation technology favorably affected the size of the specific surface area as well as volume and surface area of mesopores in the case of obtained zeolite-based sorbent A-1 and B-1, compared with the starting raw material.

## 2. Materials and Methods

### 2.1. Materials

Clinoptilolite dust (zeolite dust) obtained during the processing of Miocene volcanic tuffs from two Transcarpathian deposits located in Ukraine (Sokyrnytsya deposit) and Slovakia (Nižný Hrabovec Mine) was used for experimental studies involving the production of agglomerates for the sorption of petroleum substances. A detailed mineralogical-petrographic characterization of the dusts was presented by the authors in a previous study [38]. Within the framework of the present study, the dusts were examined for basic physical and mechanical properties such as shape, grain size distribution, specific surface area (by low-temperature nitrogen sorption and desorption, Blain’s method), bulk density and moisture content. Such technological parameters of the dusts as bulk and shaken density were also determined. The dusts were then subjected to a two-stage pressure agglomeration process using three different binding agents, as will be discussed later. As a result, six different agglomerates were produced, which were tested for use as a sorbent of oil substances by determining textural parameters, water and oil absorption, grain size, bulk density, discharge strength, and abrasion resistance. In addition, microscopic observations were made of the structure of the obtained agglomerates. For comparison of sorption properties and basic physical-mechanical properties, a commercial sorbent of petroleum substances produced from calcined diatomite—DAMSORB (Producer Imerys Industrial Minerals, Nykobing Mors, Denmark) with a grain size of 0.5–1 mm—was used [39].

### 2.2. Methods

The shape of the dust particles was determined by the descriptive method according to EN ISO 3252 [40] using a Nikon SMZ 1000 series stereo microscope with magnification ranging from 4× to 480× and a field emission scanning SEM microscope (FEG, Warsaw, Poland). The microscopes were also used to analyze the structure of the zeolite agglomerates produced. The grain size of the dust and the binders used in the agglomeration process were determined by laser diffraction with a Malvern Mastersizer 2000 analyzer using the dry dispersion method according to ISO 13320 [41]. The grain size of the zeolite agglomerates produced was determined by sieve analysis according to EN 933-1 [42]. Textural parameters of clinoptilolite dust and the agglomerates produced from it were determined by the method of isotherms of low-temperature nitrogen adsorption and desorption at −196 °C. The analysis was performed using Micromeritics’ ASAP 2020 apparatus in the relative pressure range from about 10^−3^ to 0.99. The samples were subjected to a drying process at 150 °C before measurement. Specific surface area (S_BET_) was determined by the Brunauer, Emmett, and Teller (BET) multilayer adsorption method, total pore volume (V_TOT_^0.99^) was determined by the t-method and the Barrett, Joyner, and Halenda (BJH) method, micro pore area was determined by the t-method, and average pore diameter (D_p_) was determined by the BJH results. The specific density of the dust was determined using the pycnometric method according to EN 1097-7 [43], while the bulk density and compacted density according to EN 1097-3 [44]. The moisture content of zeolite dust, agglomeration process feedstock, and moldings were determined using a weighing machine by drying at 105 °C to constant weight. The susceptibility of zeolite dust to pressure agglomeration by the two-stage method was determined by sieve analysis by determining the content of the 0.5–2.5 mm fraction in the agglomeration process product. The gravitational drop strength of the produced agglomerates was determined by dropping a sample of about 50 g three times from a height of 1 m onto a steel plate. The shedding strength is determined by the ratio of the weight of the agglomerate after sifting on a sieve with a mesh size corresponding to half the lower dimension of the agglomerate (a 0.250 mm sieve was used in this work) to the weight of the sample before the shedding test.

The drop strength was determined according to the formula:

K = A_R_/A × 100% (1)

where:

K—agglomerate’s resistance to gravitational dropout (%)

A—weight of sample before dropping (g)

A_R_—weight of the sample which is left on the sieve (g)

Abrasion resistance was determined by the author’s method using a Bond mill. The method consisted of subjecting 40 g material to 50 cycles of abrasion with the drum revolutions at 71 per minute in a mill without loading balls. Abrasion resistance is determined by the ratio of the weight of the agglomerate passing through a control sieve with a mesh size of 0.5 mm (for the purposes of this paper, this value was established) to the weight of the weight used in the test. Abrasion resistance is determined by the percentage of agglomerate that is reduced to a grain size of less than 0.5 mm during the abrasion process. The amount of abrasion resistance was determined according to the formula:

A_B_ = (m_1_ − m_2_)/m_1_ × 100% (2)

where:

A_B_—Bond mill abrasion resistance (%)

m_1_—weight of the sample put through the abrasion process (g)

m_2_—weight of sample left on 0.500 mm sieve (g)

The absorbency of the agglomerate against oil substances was determined by the Westinghouse method of determining the absorption capacity of light fuel oil by the sorbent. The test is performed in a cone with a diameter of 70 mm and a height of 75 mm made of stainless steel mesh with a mesh of 0.250 mm. A sample of 20 g is placed in the cone and then in a dish filled with oil and left for 10 min, after which time the funnel with the sample is removed from the vessel is set aside for 5 min and then weighed. In the study, Verva On diesel fuel (Polish petrol stations network Orlen) with a density of 0.820–0.845 g/cm^3^ at 15 °C was used [45]. The absorbency of the sample due to the oil is determined according to the formula:

R_oil_ = (m_2_ − m_1_)/m_1_ × 100% (3)

where:

R_oil_—oil sorption capacity (% wt)

m_1o_—weight of the sample dried to a constant mass before testing (g)

m_2o_—weight of sample saturated with oil (g)

The Westinghouse method was adapted for the determination of water absorption by the agglomerate by using distilled water instead of oil. The entire test procedure was the same as for the determination of absorbency against oil. The absorptivity of the sample due to water was determined according to the formula:

R_water_ = (m_2w_ − m_1w_)/m_1w_ × 100% (4)

where:

R_water_—water sorption capacity (% wt)

m_1w_—weight of the sample dried to a constant mass before testing (g)

m_2w_—weight of sample saturated with water (g)

All of the equipment except for the ASAP 2020 Micromeritic instrument (ASAP 2020, Micromeritics, Cracow, Poland) used in tests is in possession of the Łukasiewicz Research Network ICiMB Cracow. The nitrogen adsorption/desorption tests with ASAP 2020 from Micromeritic were conducted in the Organic Technology Laboratory Faculty of Chemistry Jagiellonian University in Cracow.

## 3. Agglomeration Experiments

**Figure 1:**
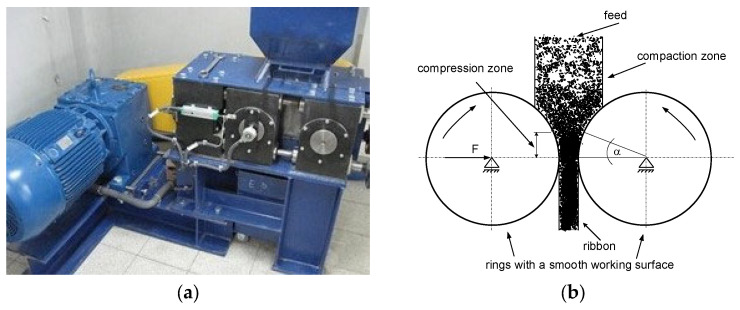
Laboratory-scale high pressure grinding roll HPGR (**a**) and press layout (**b**).

**Figure 2:**
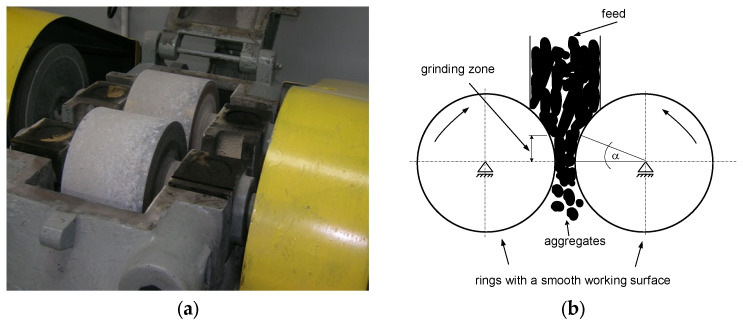
Laboratory-scale roll crusher (**a**) and crusher layout (**b**).

**Figure 3:**
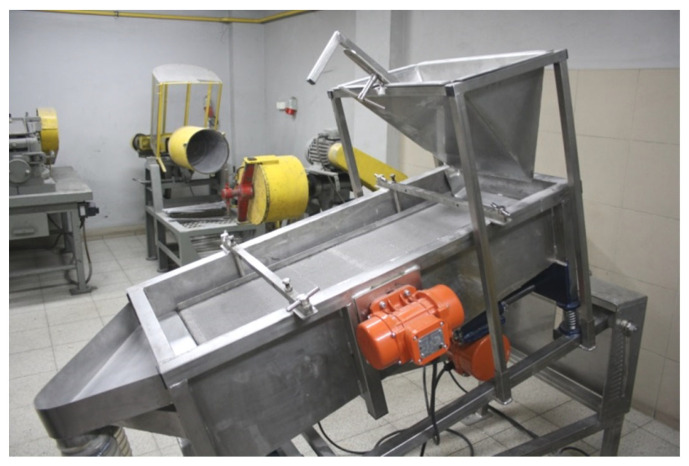
Laboratory-scale vibrating screen.

The pressure agglomeration process was carried out in a two-station system consisting of a HPGR high-pressure grinding roller with smooth-surface forming rings with a roller working diameter of 0.30 m (Figure 1) and a roller crusher with a gap width of 4 mm (Figure 2) and a vibrating screen (Figure 3). The consolidation process was carried out with constant parameters of the roller press: speed of the rollers 0.1 m/s, roller force 150 kN, gap width between the working surfaces of the rollers 4 mm. The roller press was equipped with a gravity feed. Earlier studies on zeolite dust agglomeration by the pressure method [46] showed that the optimal moisture content of the feed should be between 20 and 22% wt. It was also found, that in addition to the wetting agent (water), the process of zeolite dust agglomeration by the pressure agglomeration method requires the use of a suitable binding agent, which is typical for most dusty materials [46,47]. At this point, it is worth noting that the physical and chemical properties of the materials fed to the agglomeration and the agglomeration method used have a significant impact on the formation of the textural properties of the produced sorbents and this affects, among other things, the sorption properties and durability of the agglomerates [48]. In addition, the parameters of the agglomeration process also shape the properties of the obtained products [49].

**Figure 4:**
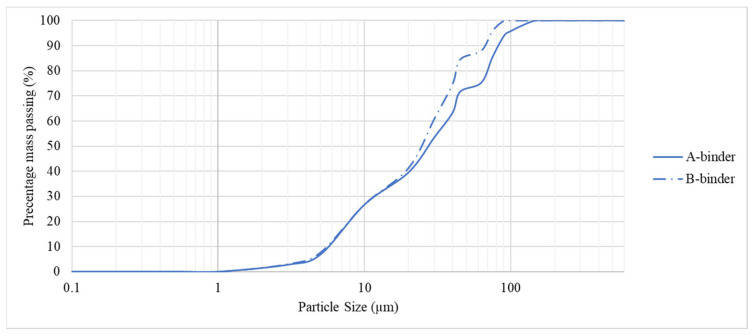
Particle size distribution curves of A-binder and B-binder used in the compaction process.

Experimental studies on the formation of zeolite agglomerates tested the effectiveness of three different binders (A, B, C), the names of which were coded due to patent procedures. Binding agent A and B are solid materials, while binder C is a liquid. Figure 4 shows the grain size distribution of the solid binding agents. Binding agent A has grain sizes ranging from 0.2 to 100 µm with the predominant grain size class above 5 µm accounting for about 93% of the total particle population. The most common particle size is 47 µm. Binder B has a grain size of 0.2 to 90 µm with a predominance of grains above 5 µm that make up about 92% of the total particle population. The most common particle size is 29 µm. The results obtained indicate similar grain size parameters of the binders used. However, the grain size of the binders is different from the grain size of the zeolite dust subjected to the consolidation process, as will be discussed later in the article.

Table 1 shows the compositions of the feeds for the pressure fusion process, as well as the moisture content of the material before the integration process and the moisture content of the ribbons.

**Figure 5:**
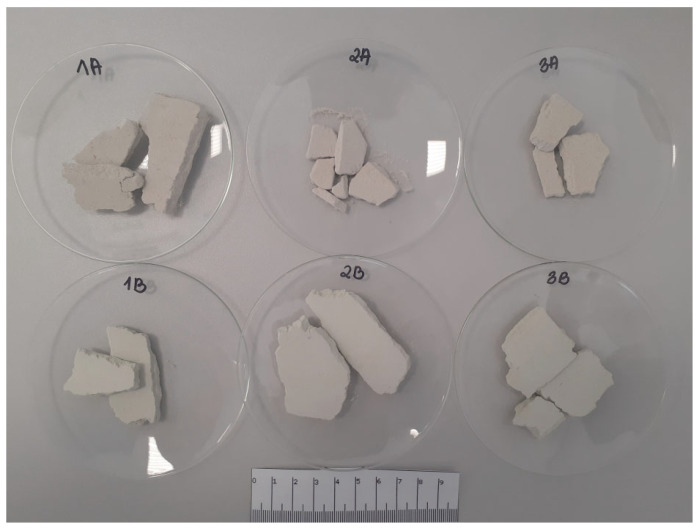
Produced ribbons (first step of the dry agglomeration).

**Figure 6:**
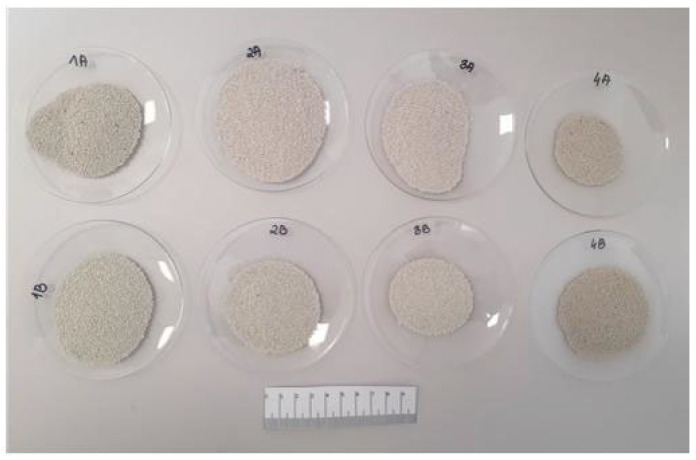
Produced agglomerates (second step of the dry agglomeration).

The first stage of agglomeration consisted in obtaining pieces of material in the form of different sized moldings with an average thickness of 6 mm (Figure 5) using an HPGR high-pressure grinding roller. The next step was to season the moldings under appropriate conditions adapted to the type of binder used. The molded material with binder A was seasoned under high humidity conditions (RH ≥ 95%) at room temperature of about 20 °C for 7 days, and then dried at 105 °C for 24 h. B and C binder moldings were seasoned at 105 °C for 24 h. The compressed material was then crushed in a roller crusher and grain classification was carried out using a vibrating screen to separate two grain classes of 2.5–1 mm and 1–0.5 mm. In further studies, the 1–0.5 mm fraction was used (Figure 6) because most commercial oil sorbents have a grain size of 0.5–1 mm. The 2.5–1 mm fraction will be the subject of another paper.

## 4. Results and Discussion

### 4.1. Results of Research on Clinoptilolite Dust (Zeolite Dust)

**Figure 7:**
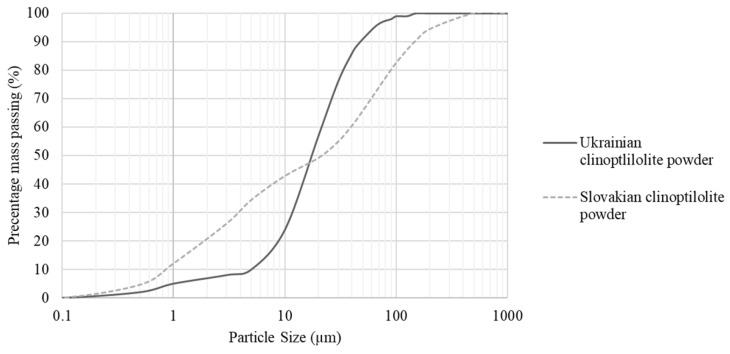
Particle size distribution curves of Ukrainian and Slovakian clinoptilolite powders used in the compaction process.

Investigations of the shape of dust particles subjected to agglomeration by the two-stage method showed that the two materials studied have the same shape—globular shape according to EN ISO 3252 [40]—while they differ in terms of particle size distribution (Figure 7). Ukrainian dust has a grain size range from 0.2 to 125 µm with the dominant grain class above 10 µm accounting for about 76% of the total particle population. The most common particle size is 18 µm. Slovak dust has a grain size ranging from 0.2 to 350 µm with a predominance of grains above 3 µm that make up about 74% of the total population. The most common particle size is 64 µm. The difference in the grain size of the materials can be seen in the content of the fraction below 5 µm, which is about 34% for Slovak dust and about 10% for Ukrainian dust. The grain size of dust from Ukraine and Slovakia more or less deviates from the particle size of the binding agents (binding agent A and binding agent B) used in the pressure consolidation process (Figure 4). Ukrainian dust in terms of grain size distribution shows more similarity to binder B. Slovak dust, in terms of particle size, is more compatible with binder A. According to Panek et al. [48], the compatibility of the grain size of the material subjected to the consolidation process with the grain size of the binder is an important factor affecting the properties of the obtained products. The similar grain size of the materials results in agglomerates with higher integral density.

**Figure 8:**
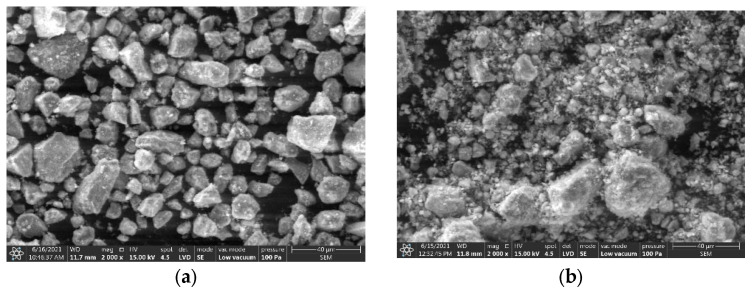
SEM images of Ukrainian clinoptilolite powder (**a**) and Slovakian clinoptilolite powder (**b**).

Observations in the micro-area (SEM) showed that the dust from Ukraine is dominated by shaped particles with sharp edges, while the dust from Slovakia is dominated by rounded particles (Figure 8a,b). The morphology of clinoptilolite dust is influenced by the mineralogical composition of the raw materials from which it was produced, i.e., volcanic tuff from Ukraine and Slovakia. The mineralogical composition of the raw material shapes its physical and mechanical properties, among others, resistance to mechanical processing and, consequently, the shape, size, and size distribution of the particles. In turn, dust with different grain size and particle shape in the process of fusion can give a product (agglomerate) with different strength properties and physicochemical parameters (including sorption properties) [48]. However, it should be noted that the properties of the agglomeration product depend not only on the properties of the material being agglomerated but also on the parameters of the entire process regardless of the agglomeration method used [49,50].

Mineralogical studies, which were the subject of another paper by the authors [38], showed that in addition to clinoptilolite, which is the main mineral component of the raw materials, minerals such as quartz and feldspars, minerals from the mica group, and cristobalite, which was identified only in the dust from Slovakia, were present in the samples. In addition, dust from Ukraine was found to contain a lower concentration of clinoptilolite and a higher quartz content compared to dust from Slovakia [38]. This affects the physical and mechanical properties of the final product including, among other things, grain size [50,51]. The higher silica content in the tuff from Ukraine hinders the disintegration process during the processing of the raw material, causing more wear on the working elements of the machines.

**Figure 9:**
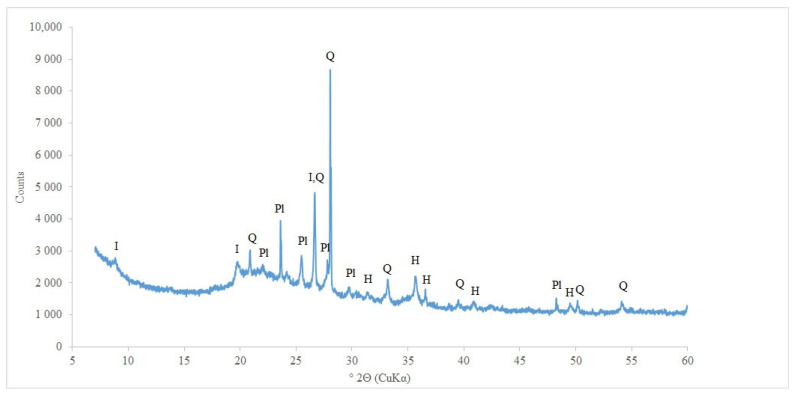
XRD pattern of commercial oil adsorbent (I—illite, Q—quartz, Pl—plagioclase, H—hematite).

X-ray analysis of the commercial sorbent (Figure 9) showed that amorphous silica, plagioclase, illite, and hematite are present in its mineralogical composition. The presence of amorphous silica is evidenced by the raised background of the diffractogram in the region of about 22° 2Θ and SEM observations, which will be discussed later in the paper.

Table 2 shows the results of tests of the basic physical and mechanical properties of dusts obtained from crushing clinoptilolite tuffs. The higher specific surface area of Slovak dust compared to Ukrainian dust is due to the higher concentration of clinoptilolite in this raw material.

### 4.2. Experimental Results

**Figure 10:**
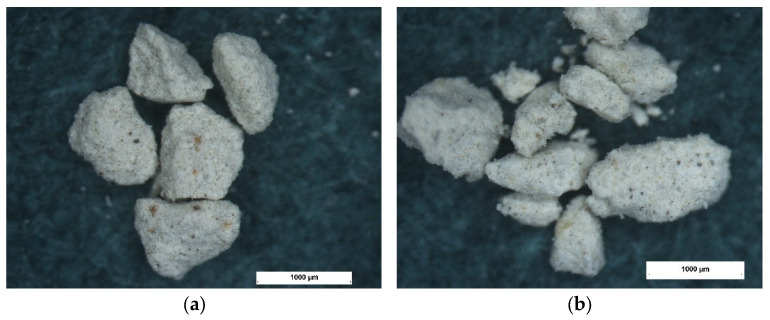
Microscope pictures of A-1 agglomerate (**a**) and A-2 agglomerate (**b**).

**Figure 11:**
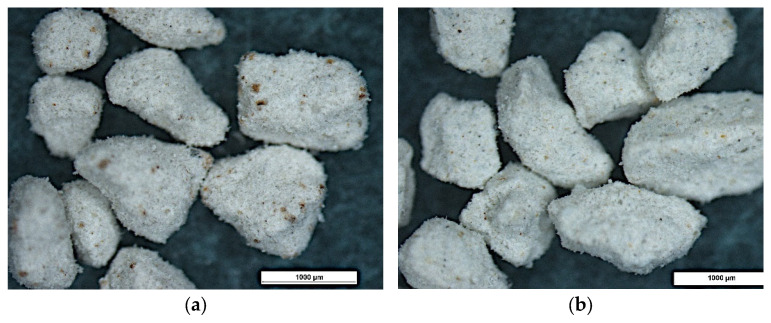
Microscope pictures of B-1 agglomerate (**a**) and B-2 agglomerate (**b**).

**Figure 12:**
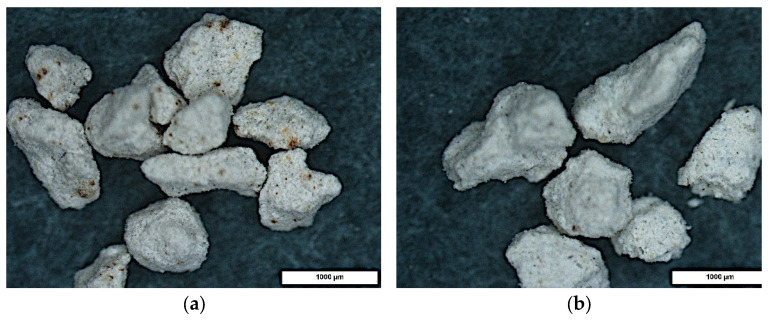
Microscope pictures of C-1 agglomerate (**a**) and C-2 agglomerate (**b**).

**Figure 13:**
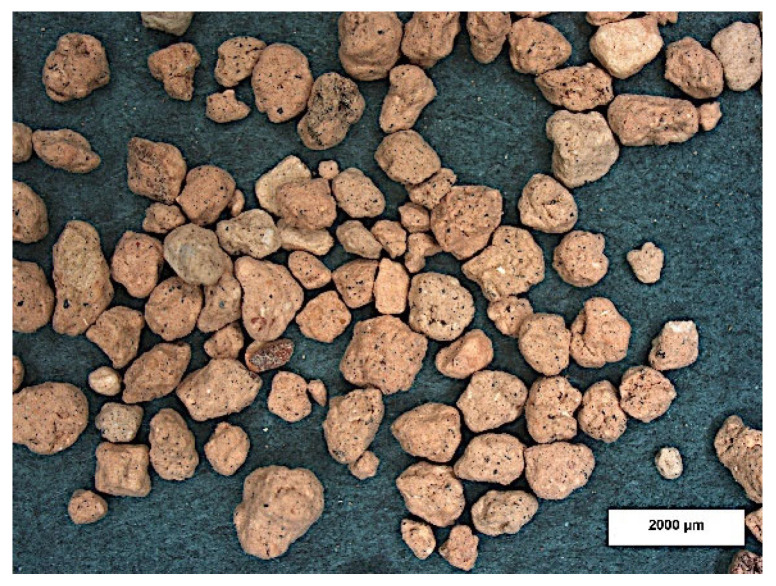
Microscope picture of commercial sorbent.

Microscopic examination using a stereoscopic microscope showed little difference in morphology between the different types of granules tested but significant differences compared to granulated commercial sorbent. The granules produced are angular in shape with sharp edges and a rough surface (Figure 10, Figure 11 and Figure 12). In contrast, granulated commercial sorbent is mainly ellipsoidal, spherical, and rounded grains with smooth edges and surface (Figure 13).

**Figure 14:**
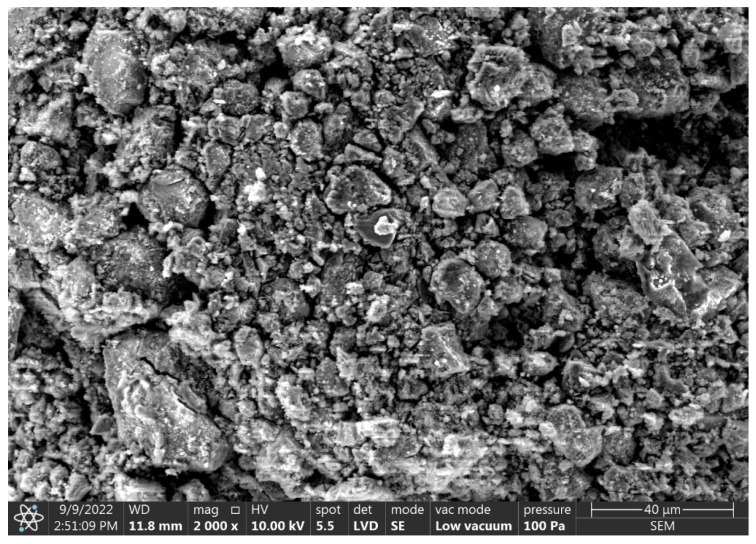
SEM images of the C-1 agglomerate surface.

**Figure 15:**
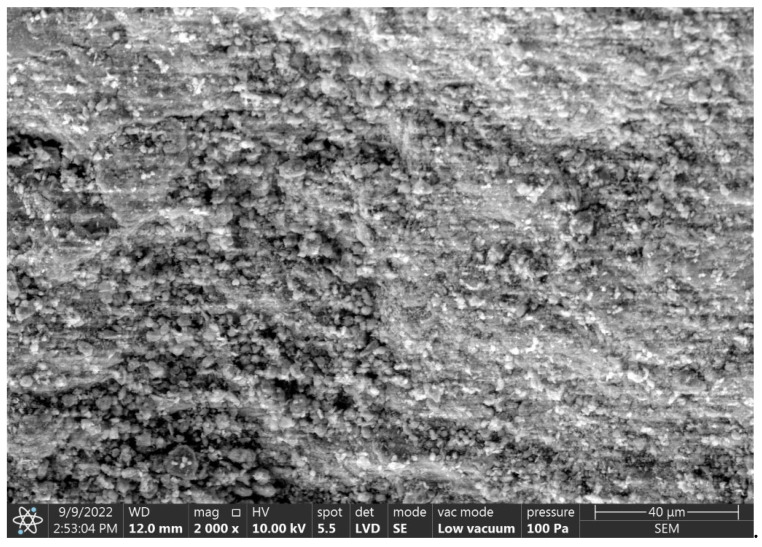
SEM images of the C-2 agglomerate surface.

**Figure 16:**
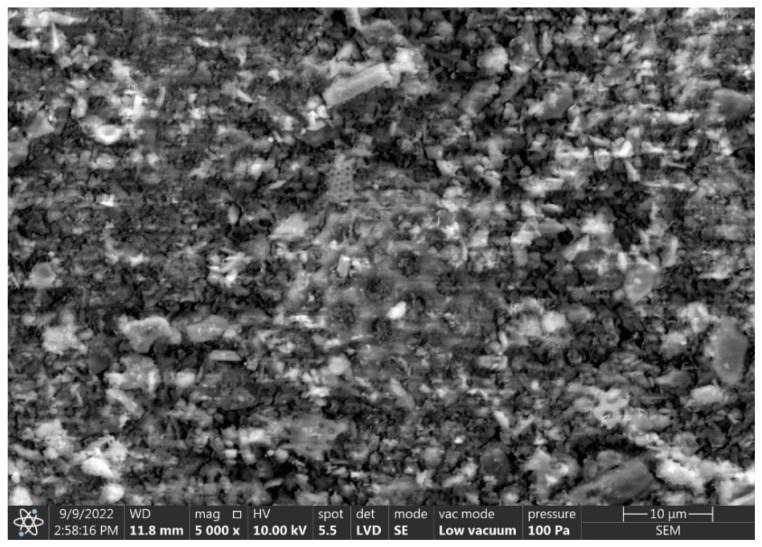
SEM images of the commercial sorbent (diatom shell in the center).

Some difference in the morphology of the produced granules can be seen in the degree of surface roughness and homogeneity of microstructure in SEM observations (Figure 14, Figure 15 and Figure 16) depending on the origin of the clinoptilolite dust. The surface of clinoptilolite dust agglomerates from Ukraine (Figure 14) is more homogeneous and characterized by higher roughness and larger pore sizes compared to the surface of clinoptilolite dust agglomerates from Slovakia (Figure 15). The size of the pores has an impact on the absorbency of petroleum substances, which are characterized by large particle sizes. The larger pore size of the Ukrainian granules may suggest better absorption of oil substances in this case.

Comparing the microstructure of the produced sorbents and the commercial sorbent, it can be concluded that the surface of the Ukrainian dust granules is more developed. The surface of the Slovak dust granules and the commercial sorbent is similar and less developed. In addition, SEM observations of the microstructure of the commercial sorbent identified remnants of diatom carapaces (Figure 16).

**Figure 17:**
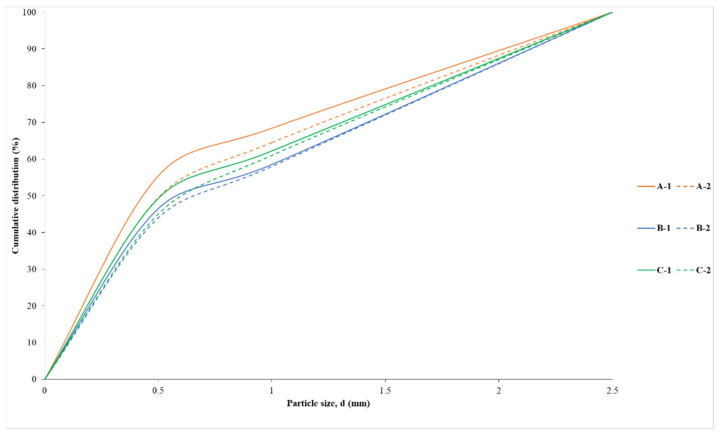
Efficiency of the dry agglomeration processes.

The efficiency (Figure 17) of the agglomeration process using the two-stage method for all samples is very similar and averages 50%, only in the case of clinoptilolite dust from Ukraine with binder A, the efficiency of the process is slightly lower at 45%, while for the same binder used to agglomerate clinoptilolite dust from Slovakia, the efficiency of the process is already 50%. The best agglomeration process efficiency was found when binder B was used and is 56% and 53% for dust from Slovakia and Ukraine, respectively.

**Figure 18:**
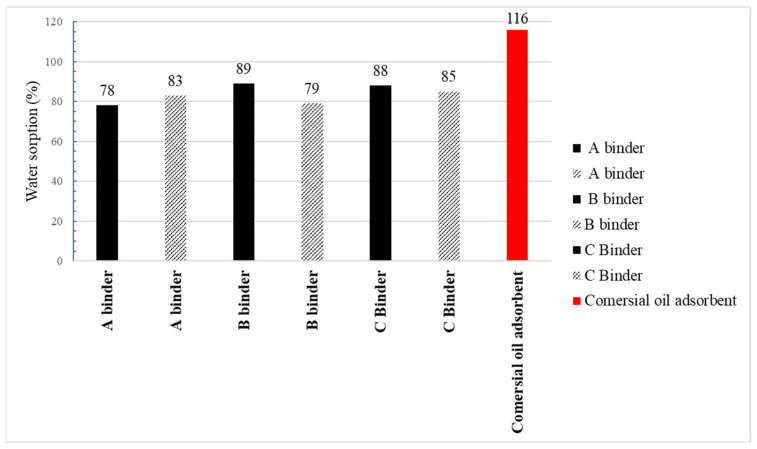
Water sorption agglomerates and commercial adsorbent.

**Figure 19:**
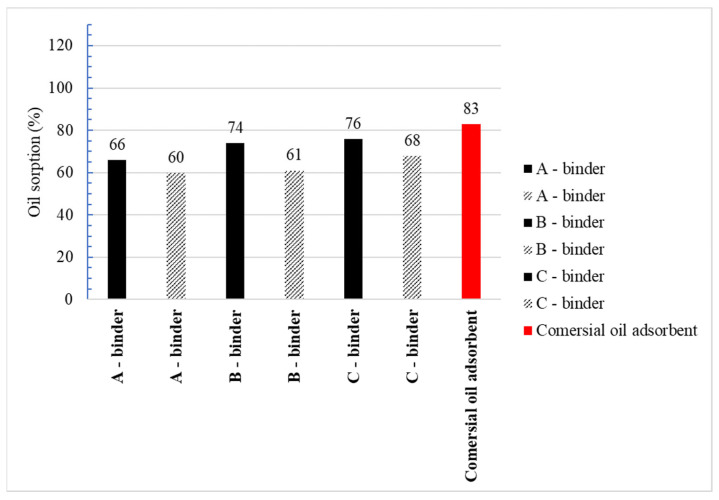
Oil sorption agglomerates and commercial adsorbent.

Figure 18 and Figure 19 show the results of the absorbency test of water and oil substances using the Westinghouse method. The minimum required absorbency to petroleum substances of sorbents approved for use by PPS units in Poland according to the Journal of Laws of 2010. No. 85, item 553 [52] is 50% by weight of the sorbent. The study shows that all the clinoptilolite granules produced by the two-stage agglomeration method achieve the required absorbency value against oil substances.

The highest absorbency is shown by granules obtained from clinoptilolite dust from Ukraine with binder C for which the determined oil sorption was 76% and water sorption was 88%. Comparable parameters were obtained for granules of the same origin with binder B. In the case of granules made from Slovak dust, the best absorption was obtained with binder C, but oil sorption is about 11% less. The study shows that the use of binder C in the agglomeration of clinoptilolite dust from Ukraine and Slovakia gives the best results in terms of sorption properties. At the same time, Ukrainian dust is a better sorbent. This suggests a higher proportion of mesopores in the microstructure of Ukrainian dust granules compared to the microstructure of Slovak dust granules. Petroleum substances are liquids with high viscosity and large particles therefore they can only be absorbed through pores of appropriate size [5]. Comparing the sorption properties of the produced granulate with the best absorbency with the commercial sorbent, it can be concluded that the water absorbency for the produced granulate is lower by about 22%, and the oil absorbency by about 8%. The better sorption properties of the commercial sorbent are related to the use of the calcination process in its manufacture. The calcination process improves the sorption properties and imparts greater mechanical resistance to the granules at the expense of high energy expenditure and the release of harmful substances into the atmosphere during its production [53].

The kinetic strength determined by the gravitational shedding method of all produced pellets is similar and satisfactory (Table 3). The produced pellets, except for the granulate made from Ukrainian dust with binder B, achieved a shedding strength of at least 90%, which, according to literature data, characterizes good quality briquettes due to transport and storage regardless of shape and size [54,55]. The highest gravitational discharge resistance was achieved by clinoptilolite dust pellets from Ukraine with binder C 95% and is 3% lower than the discharge resistance for commercial sorbent (98%). Granules made from clinoptilolite dust from Slovakia showed better abrasion resistance compared to granules made from clinoptilolite dust from Ukraine. The best comparable resistance in both cases was shown by materials with C binder (16% and 15% for dust from Ukraine and Slovakia, respectively). On the other hand, the abrasion resistance of the commercial sorbent is about 50% better than the granulate with the best abrasion resistance parameters (C-binder granulate). The results showed that both the physical-mechanical properties of the agglomerated material and the agglomeration method used affect the material’s abrasion susceptibility. It is worth noting that no such relationship was observed in the case of resistance to gravitational dropping.

The results of textural studies of starting raw materials, produced agglomerates, and commercial sorbent are presented in Table 4. In terms of specific surface area (S_BET_), it was found that both starting raw materials (clinoptilolite dust from Ukraine and Slovakia) from which agglomerates were produced and commercial sorbent have a relatively low specific surface area BET (14–28 m^2^/g). Also determined, based on t-plot analysis, the volumetric amount of micropores is small and corresponds to a small amount of adsorbed nitrogen at the lowest relative pressure (P/Po < 0.01). Clinoptilolite dust from Ukraine was characterized by about twice the specific surface area, total pore volume, and volume and surface area of mesopores compared to clinoptilolite dust from Slovakia. The above differences affect the textural parameters of agglomerates produced from these two dusts. It is noteworthy that the process of high-pressure agglomeration favorably affected the size of the specific surface area, as well as the volume and surface area of mesopores in the case of samples produced from clinoptilolite dust A-1 and B-1 compared to the starting raw material. In contrast, an inverse relationship was observed for samples of agglomerates produced from dust from Slovakia. In this case, the produced products are characterized by worse textural parameters compared to the starting raw material. Comparing the textural parameters of the granules obtained and the commercial sorbent, it can be concluded that samples B-2 and C-2 have very similar textural parameters to the commercial sorbent, indicating the effectiveness of the dust consolidation method carried out. In addition, all granules produced from clinoptilolite dust from Slovakia have a larger volume of mesopores compared to the commercial sorbent. In addition, all granules made from clinoptilolite dust from Slovakia have a higher volume of mesopores compared to commercial sorbent. Previous studies have shown that the proportion of mesopores in the total pore volume of the material and the viscosity of the oil substance play a key role in the absorption of oil substances. Bandura [1] showed a high correlation between the surface area of mesopores and the sorption capacity of sorbents. The present study did not confirm the above hypothesis and suggests that another factor shapes the sorption properties of the tested materials.

**Figure 20:**
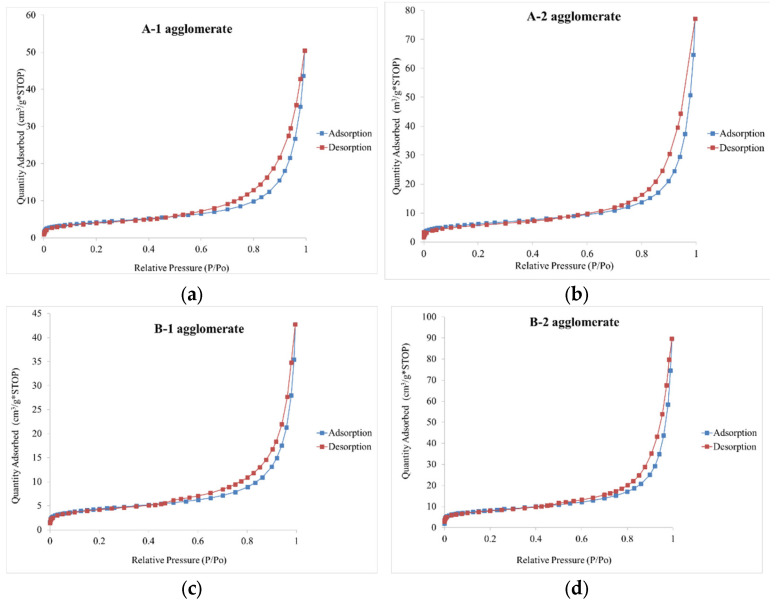

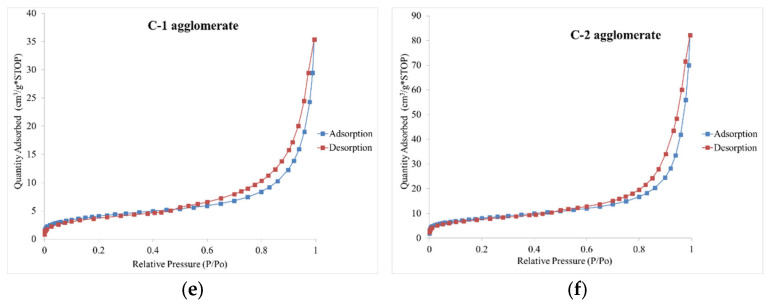
Absorption-desorption isotherms of nitrogen for the manufactured agglomerates (**a**–**f**).

**Figure 21:**
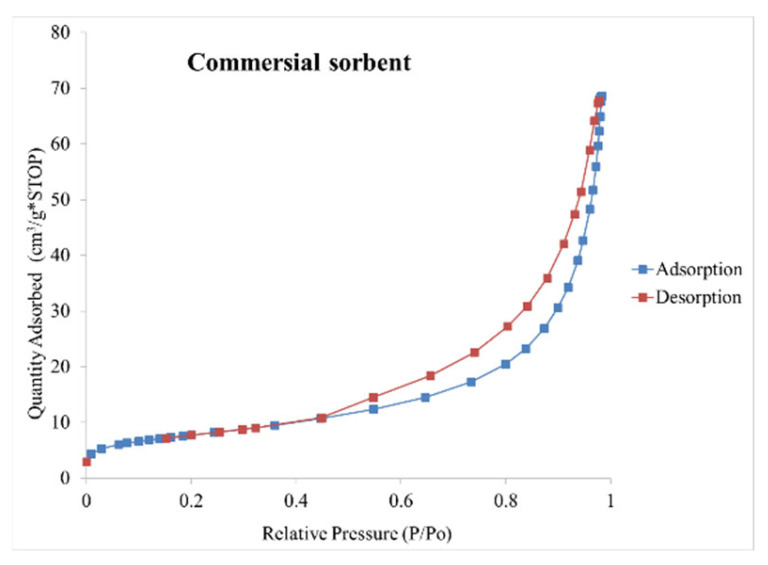
Absorption-desorption isotherms of nitrogen for the commercial sorbent.

The pore diameter in all the agglomerates produced is similar, ranging from 10 to 12 nm. The pores in the commercial sorbent are slightly wider compared to the agglomeration products, with a diameter of 15 nm, which may account for the increased sorption properties of petroleum substances by the commercial sorbent. Nitrogen adsorption and desorption isotherm curves for the produced agglomerates (Figure 20a–f) and the commercial sorbent (Figure 21) look very similar and can be classified as type II according to IUPAC [56]. On the other hand, the hysteresis loops were classified as type H3 according to IUPC [56] and type B according to [57], indicating a slit-shaped pore with a narrow so-called bottle-shaped entrance.

## 5. Conclusions

This study has proven that it is feasible to produce zeolite-based granular petroleum sorbent from waste dust by pressure agglomeration without highly energy-consuming calcination. The summary of the key findings of the research are as follows:

- All the agglomerates produced meet the requirements for oil absorbency efficiency for sorbents used by fire departments in Poland, which is 50% by weight of the sorbent.
- The optimum feed composition for presented agglomeration technology that provides the granular zeolite-based sorbent with the best properties was established: 6% of C-binder and moisture content within a narrow range of 20–20.6%.
- The effectiveness of the presented zeolite dust agglomeration method was proven by textural parameters of the obtained zeolite-based sorbents. In case of produced B-2 and C-2 agglomerates, the achieved specific surface area (S_BET_) was 28.1 m^2^/g and 28.3 m^2^/g, respectively, that closely corresponds to the specific surface area of the commercial sorbent (S _BET_ = 28.1 m^2^/g).
- Interestingly, all granules obtained from zeolite dust from Slovakia (A-2, B-2, C-2) achieved an increase of 19%, 33%, and 35% respectively, in volume of mesopores, compared to the commercial sorbent.
- Zeolite-based sorbent A-1 and B-1 achieved an increase of 17% and 18%, respectively, in specific area surface and an increase of 35% and 12%, respectively, in mesopores volume, compared with the raw material (waste dusts).
- The absorption efficiency of petroleum substances and resistance to gravitational drop test results for the best quality produced zeolite-based sorbent (C-1 agglomerate) indicated a decrease of 8% and 3%, respectively, compared with the commercial sorbent obtained in calcination process.
- As expected, the test results of abrasion resistance for produced zeolite-based sorbents are significantly inferior compared to the commercial sorbent.
- In addition, it was shown that in the samples analyzed, the sorption efficiency of petroleum substances does not correlate with the total volume of mesopores and the content of clinoptilolite, a mineral with sorption properties. On the other hand, a correlation with the shape and dimension of the pores was noted but this requires further research.
- Furthermore, the obtained results showed that the grain size of the compaction material and the compatibility of its grain size with that of the binder are more important in terms of oil absorption efficiency.
- Studies have shown that the unfavorable grain composition of the agglomeration process feedstock, which results in a decrease in abrasion resistance, can be leveled by using a suitable binder.

Finally, the outcomes of this this investigation proved that it is possible to produce the efficient oil sorbent in a much more eco-friendly and green way, compared with that of the commercial sorbent. The proposed agglomeration technology highlights a pragmatic remedial solution for reducing the greenhouse gases emissions and utilizing the waste material.

## Figures and Tables

**Table 1:** Feed composition for dry granulation, feed moisture, and ribbon moisture.

Sample	Feed Composition	Feed Moisture (%)	Ribbon Moisture (%)
A-1	Ukrainian clinoptilolite powder + 3% A-binder	21.0	15.0
A-2	Slovakian clinoptilolite powder+ 3% A-binder	22.0	16.3
B-1	Ukrainian clinoptilolite powder + 5% B-binder	20.8	18.8
B-2	Slovakian clinoptilolite powder + 5% B-binder	20.0	19.2
C-1	Ukrainian clinoptilolite powder + 6% C-binder	20.6	19.3
C-2	Slovakian clinoptilolite powder + 6% C-binder	20.0	18.0

**Table 2:** Physical properties of powder materials from Ukraine and Slovakia.

Powder Material	Specific Density (g/cm^3^)	Tapped Density (g/cm^3^)	Moisture (%)	Bulk Density (g/cm^3^)	Specific Surface S_BLAINE’A_ (cm^2^/g)	Specific Surface _BET_ (cm^2^/g)
Ukraine	0.889	1.108	4.6	2.28	3125	12.58
Slovakia	0.634	0.777	4.6	2.24	8837	29.91

**Table 3:** Physical properties of the produced agglomerates and commercial sorbent.

Binder Type	Compaction Method	Specific Density (g/cm^3^)		Drop Strength (%)		Abrasion Resistance (%)	
		Ukraine	Slovakia	Ukraine	Slovakia	Ukraine	Slovakia
A	Dry agglomeration	0.73	0.64	90	94	27	19
B		0.73	0.67	89	92	21	18
C		0.7	0.66	95	94	16	15
Commercial sorbent		0.66		98		7	

**Table 4:** The textural parameters for clinoptilolite powders and their consolidated forms using various binders.

Sample	S_BET_ (m^2^/g)	V_tot_ (cm^3^/g)	V_mic_ (cm^3^/g)	S_mic_ (m^2^/g)	V_mes_ (cm^3^/g)	S_mes_ (m^2^/g)	D_p_ (nm)
Ukrainian clinoptilolite powder	12.6	0.059	0.002	3.18	0.057	9.41	12.0
A-1	14.8	0.078	0.001	2.68	0.077	12.08	12.0
B-1	14.9	0.066	0.002	4.63	0.064	10.27	11.0
C-1	14.4	0.055	0.002	3.37	0.053	11.03	10.0
Slovakian clinoptilolite powder	29.9	0.122	0.003	5.68	0.119	24.22	11.0
A-2	21.8	0.119	0.002	4.54	0.117	17.23	12.0
B-2	28.1	0.138	0.004	7.34	0.134	20.80	12.0
C-2	28.3	0.127	0.003	5.43	0.124	22.91	11.0
Commercial sorbent	28.0	0.096	0.001	2.44	0.095	25.56	15.0

S_BET_—specific surface area, V_tot_—volume of all pores, V_mic_—volume of micropores, V_mes_—volume of mesopores (estimated as the total volume of pores minus volume of micropores), S_mic_—surface of micropores, S_mes_—surface of mesopores (external surface), D_p_—average pore diameter.

## Data Availability

The data presented in this study are available on request from the corresponding author.
